# Supplementary material for: MetaRibo-Seq measures translation in microbiomes
Source: Nat Commun. 2020 Jun 29;11:3268. doi: 10.1038/s41467-020-17081-z (PMC7324362; doi:10.1038/s41467-020-17081-z)
Supplement: Supplementary file 10 — Supplementary Data 7 [file 41467_2020_17081_MOESM10_ESM.zip › File2/Confidence_VeryHigh_Taxonomy/280054_out.krona.html]

Javascript must be enabled to view this page.

members
magnitude
magnitudeUnassigned
count
unassigned
taxon
rank

280054\_out

11

10
2759
superkingdom

kingdom
4751
10

subkingdom
10
451864

phylum
2
5204

subphylum
1
452284

1
1538075
class

162474
1
order

family
742845
1

55193
1
genus

180528
1

SRS075341\_contig\_number\_15829
species

subphylum
1
5302

class
1
155616

order
1851469
1

1
1759442
family

genus
1
5552


SRS053649\_contig\_number\_contig-100\_2763.41139
species
1
82508

phylum
4890
8

subphylum
7
147538

class

SRS058070\_contig\_number\_19864SRS077392\_contig\_number\_contig-100\_1428.80497
2
7
147550

subclass
222543
5

5125
5
order

5
159075
genus

5044
5
species

SRS013687\_contig\_number\_4269SRS023914\_contig\_number\_contig-100\_2027.162373SRS051031\_contig\_number\_contig-100\_1938.196797SRS053356\_contig\_number\_46001SRS1041145\_contig\_number\_contig-100\_2300.186373

subphylum
1
147537

1
4891
class

order
4892
1

genus
5475
1


SRS075773\_contig\_number\_37199
species
1323752
1


SRS017821\_contig\_number\_contig-100\_28203.103726
1
